# Elucidating the causal associations and mechanisms between circulating immune cells and idiopathic pulmonary fibrosis: new insights from Mendelian randomization and transcriptomics

**DOI:** 10.3389/fimmu.2024.1437984

**Published:** 2025-01-17

**Authors:** Han Yang, Xuanyu Wu, Xiang Xiao, Jiajing Chen, Xiaomin Yu, Wen Zhao, Fei Wang

**Affiliations:** Hospital of Chengdu University of Traditional Chinese Medicine, Chengdu University of Traditional Chinese Medicine, Chengdu, China

**Keywords:** circulating immune cells, idiopathic pulmonary fibrosis, Mendelian randomization, transcriptomics, scRNA analysis

## Abstract

**Background:**

Growing evidence indicates an association between circulating immune cell phenotypes and idiopathic pulmonary fibrosis (IPF). Although studies have attempted to elucidate the causal relationship between the two, further clarification of the specific mechanisms and causal linkages is warranted.

**Objective:**

We aimed to conduct a two-sample Mendelian randomization (MR) analysis with transcriptomics data analysis to elucidate the causal relationship between circulating immune cells and IPF and to explore potential biomarkers.

**Methods:**

We first explored the bidirectional causal association between IPF and immune cell phenotypes using two-sample MR analysis. Genome-wide association studies data for immune cell phenotype and IPF were obtained from publicly available databases. A standardized instrumental variable screening process was used to select single nucleotide polymorphisms (SNPs) for inclusion in the MR. Five methods represented by IVW were used to assess causal effects. Subsequently, SNP-nearest genes combined with the transcriptomics data of IPF were subjected to multiple bioinformatics analyses such as TIMER, WGCNA, functional enrichment analysis, protein-protein interaction analysis, and ROC to identify IPF biomarkers. Finally, the single-cell RNA sequencing (scRNA-seq) data was used to validate our findings by single-cell analysis.

**Results:**

The MR study identified 27 immune cell phenotypes causally associated with IPF, of which 20 were associated with a decreased risk of developing IPF and 7 were associated with an increased risk. *CTSB* (AUC=0.98), *IL10* (AUC=0.83), and *AGER* (AUC=0.87) were identified as promising biomarkers of IPF. Single cell analysis showed differences in CD14^+^ CD16^+^ monocytes, CD16^+^ monocytes and Granulocyte-monocyte progenito between the IPF group and the healthy control group. The three hub genes were highly expressed in three immune cell subsets of IPF patients. It underscores the potential feasibility of three genes as biomarkers.

**Conclusions:**

Our study demonstrates the causal associations of specific immune cell phenotypes with IPF through genetic methods and identifies *CTSB*, *IL10*, and *AGER* as biomarkers of IPF through bioinformatics analysis. These findings provide guidance for future clinical and basic research.

## 1 Introduction

Idiopathic pulmonary fibrosis (IPF) is a chronic interstitial fibrosis disease of unknown cause and the most common form of idiopathic interstitial pneumonitis (1, 2). IPF patients, whose median survival after diagnosis is only 3-5 years (3), present clinically with insidious, progressive dyspnea with or without coughing until they reach respiratory failure. Repeated damage and aberrant repair of the alveolar epitheliums are central to the pathogenesis, and histopathology is characterized by honeycomb and patchy scarring (4). The global incidence and diagnosis of IPF are increasing due to changes in lifestyle and diagnostic criteria (5). Although several studies have attempted to discover the true pathogenesis of IPF, the exact mechanism of IPF has not been elucidated to date. Early detection and identification of the causative factors and pathogenesis of IPF are key to its prevention and treatment.

Immune cells are involved in the innate and adaptive immune processes of the organism. Their role in lung diseases, including pneumonia, chronic obstructive pulmonary disease (COPD), asthma, and lung cancer (6), has garnered increasing attention in recent years due to their critical impact on the onset, progression, and prognosis of these conditions. The dynamic progression and severity of lung diseases are highly dependent on antigen exposure, patient susceptibility, and the type of immune response. Immune cell-driven inflammation and related responses are strongly associated with IPF (7), with chronic inflammation driven by immune cells persisting in most patients (8). Abnormal immune-inflammatory responses *in vivo* can be induced by external environmental exposures or dysregulated cellular senescence (5). Foreign bodies and dead or dying cells activate the innate immune response, recruiting circulating immune-inflammatory cells and inducing an imbalance in autophagy and efferocytosis. Furthermore, through lymphocyte-mediated activation of mesenchymal stromal cells, they initiate a cascade of fibrotic events (9). Immune cells in fibrotic areas may also interact with alveolar epithelial cells and fibroblasts, either accelerating or slowing down the fibrotic process (10). Studies have shown a negative correlation between circulating innate lymphoid cells (ILCs) and the prognosis of patients with IPF. TGF- β1, a crucial marker of fibrosis, induces ILC activation (11), subsequently stimulating an increase in the expression of IL2, IL13, and IL25 in the lung, thereby mediating immune disorders and inflammatory damage (12). The macrophages have the plasticity, undifferentiated macrophages (M0) can be polarized in to two types: classically activated macrophages (M1) and alternatively activated macrophages (M2), both are implicated in the pathogenesis of IPF. The classically activated response releases interferon γ, tumor necrosis factor α, and IL-1β. These cytokines induce M1 polarization and pro-inflammatory effects, leading to lung parenchymal damage, pulmonary myofibroblast differentiation, and epithelial-mesenchymal transition (EMT), all of which are implicated in the pathogenesis of IPF (13). Conversely, the alternatively activated macrophages immune response releases IL-4 and IL-13, inducing M2 polarization and exerting pro-fibrotic effects.

Despite repeated emphasis on the role of immune cells in IPF, the causal relationship and underlying mechanisms between the two require further clarification. A Mendelian randomization (MR) study (14) demonstrated a significant causal relationship between 37 types of immune cells and IPF. However, this study used a small sample size of genome-wide association studies (GWAS) for IPF, and it was a unidirectional study of the causal effect of immune cells on IPF without reverse validation of the causal effect of IPF on immune cells. Another MR study (15) explored the bidirectional causal relationship between immune cells and IPF but did not further investigate the genetic overlap, susceptibility gene sharing, and deeper mechanisms between the two. Thus, we aimed to clarify the bidirectional causal relationship between circulating immune cells and IPF using a two-sample, bidirectional MR method. We sought to elucidate potential shared targets and mechanisms of immune cells mediating IPF by combining transcriptomics data with various bioinformatics methods. Our study will serve as a valuable reference for further investigations into the biomarkers and pathological mechanisms of IPF.

## 2 Materials and methods

### 2.1 Study design

**Figure 1:**
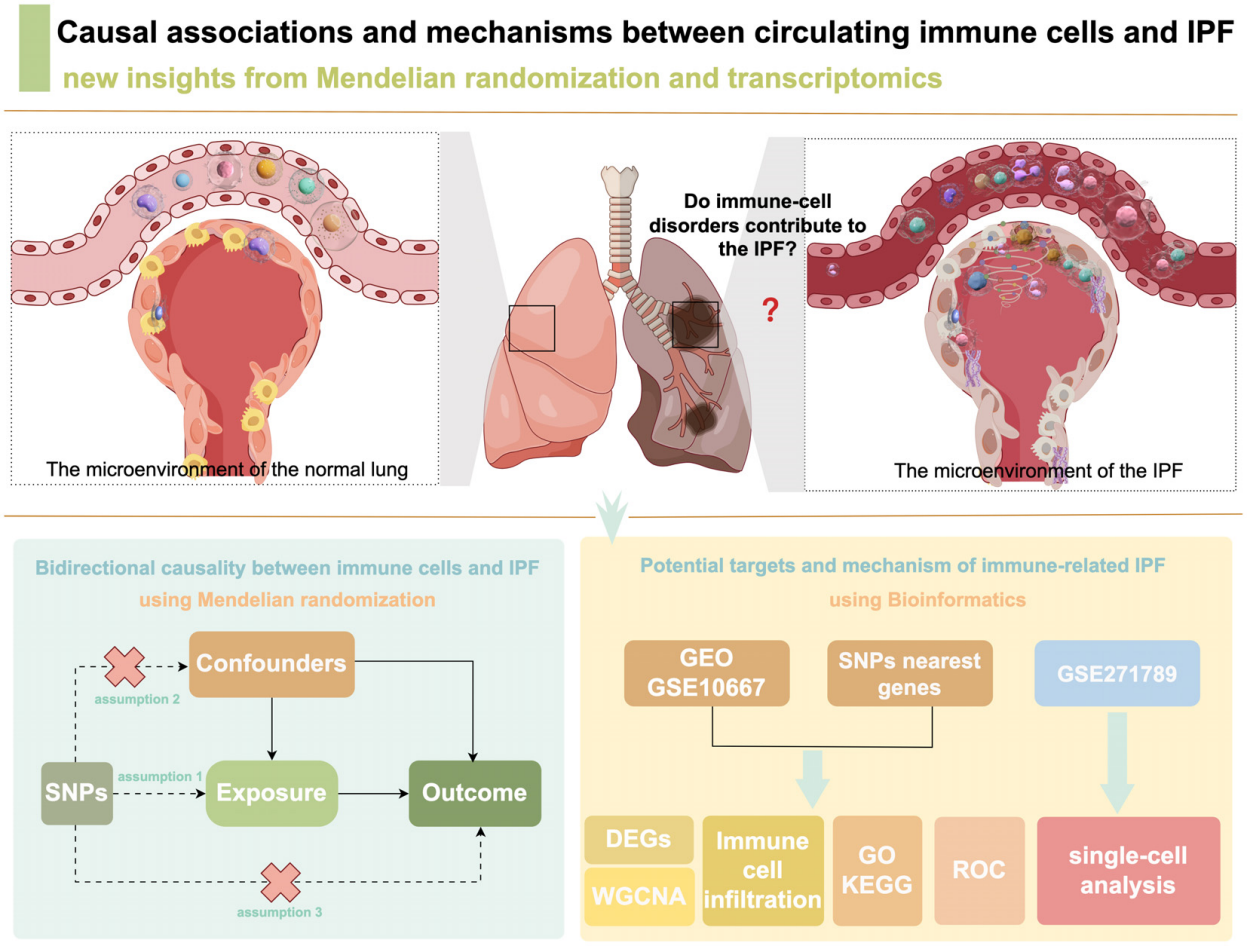
Study flowchart.

A two-sample MR analysis was conducted to assess bidirectional causal associations of 731 circulating immune cell phenotypes with IPF. The MR analysis was based on instrumental variables (IVs) as the core principle, requiring adherence to the following assumptions: (1) direct correlation between the IVs and exposure factors; (2) absence of correlation between IVs and confounders affecting the relationship between exposure and outcome; and (3) absence of IV influence on the outcome through pathways other than exposure. Bioinformatics techniques were used to identify potential biomarkers of IPF, and the expression of potential biomarkers was validated using an external dataset (**Figure 1**).

### 2.2 Two-sample bidirectional MR

#### 2.2.1 Data sources

GWAS summary data for circulating immune cell phenotypes were obtained from the GWAS Catalog (https://www.ebi.ac.uk/gwas/; GCST0001391 to GCST0002121). In total, 731 circulating immune cell phenotypes were obtained (16), namely 118 absolute cell counts, 192 relative cell counts, 389 median fluorescence intensities reflecting surface antigen level, and 32 morphologic parameters. Specifically, the data comprised seven immune cell types, namely T cells, B cells, dendritic cells (DCs), monocytes, other myeloid cells, natural killer (NK) cells, and regulatory T cells (Tregs). GWAS summary data for the IPF were obtained from FinnGen_R9 (https://www.finngen.fi/en/access_results), involving 375,082 European participants, including 2018 IPF patients and 373,064 controls.

#### 2.2.2 Selection of IVs

To ensure the validity of MR analysis results, we used a standardized process to include IVs: (1) significant correlation of IVs with exposure factors (*P*<1×10^-5^); (2) removal of chained unbalanced single nucleotide polymorphisms (SNPs) with thresholds of r=0.001 and kb=10,000; (3) exclusion of SNPs with *MAF*<0.01; (4) calculation of *F* statistics to eliminate weak IVs, where $F=\left(N-2\right)\times \frac{R^{2}}{\left(1-R^{2}\right)}$ and $R^{2}=2\times \left(1-EAF\right)\times EAF\times {\left(\frac{\beta }{SE\times \sqrt{N}}\right)}^{2}$ , with only SNPs having F>10 retained to ensure statistical efficacy; (5) removal of SNPs associated with potential confounders (aging and smoking) through the PhenoScanner V2 database (http://www.phenoscanner.medschl.cam.ac.uk/) with parameters set to *P*=1e-5, Proxies=None, r^2^ = 0.8, and Build=37; and (6) elimination of palindromic SNPs with intermediate allele frequencies.

#### 2.2.3 Statistical analysis

All data analyses were conducted using R software (V4.3.1). Inverse variance weighted (IVW) was the primary MR analysis method. Weighted median, simple mode, MR-Egger, and weighted mode analyses were performed to assess the robustness of IVW. Cochrane's *Q* test was used to test for heterogeneity among SNPs, while MR-Egger intercept was used to assess horizontal pleiotropy. Leave-one-out (LOO) was used to analyze the sensitivity and MR-PRESSO was employed to test for outliers. As the outcome indicators were dichotomous variables, the results were expressed as odds ratio (*OR*) and 95% confidence interval (95% *CI*). *P*<0.05 was considered to indicate statistically significant differences.

#### 2.2.4 Selection of SNP-nearest genes

The GWAS catalog was used to obtain the SNP-nearest genes, setting a genome-wide significance level of *P*<5×10^-8^ and harmonizing to ensure consistency in the effect of exposure concerning the effector allele and subsequent analysis.

### 2.3 Bioinformatics of immune cells mediating IPF

#### 2.3.1 Acquisition of microarray data

Transcriptomics data for IPF were obtained from the GEO database (http://www.ncbi.nlm.nih.gov/geo/). Data inclusion criteria were as follows: (1) presence of both IPF patients and normal controls in the microarray data; (2) sampling from lung tissues; and (3) species being human. The GSE10667 microarray dataset was obtained based on the criteria. It contained transcriptomics data from 8 cases of IPF lung tissue and 15 cases of normal lung tissue.

#### 2.3.2 Extraction of shared genes

Transcriptomics analysis of differentially expressed genes (DEGs) was conducted using the R package “limma” with thresholds set to “adjusted *P*<0.05 and log2(FC)>1.3 or log2(FC)<-1.3.” Expression heatmaps were plotted using the R package “pheatmap”. Subsequently, the overlap of DEGs with SNP-nearest genes was extracted by plotting a Venn diagram to identify shared genes of circulating immune cell variants mediating IPF for subsequent analysis.

#### 2.3.3 Analysis of immune cell infiltration

TIMER 2.0 (http://timer.cistrome.org/) with the “Immune Estimation” module was used to analyze the aforementioned shared genes for the degree of immune cell infiltration. It included the calculation of the infiltration of six types of immune cells, namely cluster of differentiation (CD)4^+^ T cells, CD8^+^ T cells, B cells, neutrophils, macrophages, and myeloid DCs.

#### 2.3.4 Weighted gene co-expression network analysis (WGCNA)

Gene expression profiles were used to calculate the median absolute deviation of each gene, excluding the smallest 25% of genes. Hierarchical clustering was performed using the “goodSamplesGenes” function of the “WGCNA” software package to eliminate outlier genes and samples. The “pickSoftThreshold” function was used to determine the appropriate soft threshold. Subsequently, the correlation matrix was converted to an adjacency matrix and further processed into a topological overlap matrix. The dynamic tree-cutting method was to identify each module, and the relationship between these modules and the IPF was further investigated. Finally, the module with the largest Pearson correlation coefficient was selected for further investigation.

#### 2.3.5 Functional enrichment and protein-protein interaction (PPI)

The module genes most strongly correlated with IPF were selected for the Gene Ontology (GO) function enrichment analysis and Kyoto Encyclopedia of Genes and Genomes (KEGG) pathway enrichment analysis using the Metascape (https://metascape.org/) platform. PPI network analysis of the module genes was performed using STRING (https://cn.string-db.org/), with a minimum required interaction score set to 0.4. The PPI network was visualized using Cytoscape. Finally, the top ten ranked genes were identified using five algorithms of the Cytohubba plugin, namely Degree, MCC, DMNC, EcCentricity, and Closeness. The overlapping genes were considered hub genes.

#### 2.3.6 Receiver operating characteristic (ROC) curve analysis

Based on the expression of hub genes in IPF and normal lung tissues, we generated a ROC curve using the Sangerbox online platform (http://sangerbox.com/home.html) (17). The diagnostic efficacy of the hub genes for IPF was evaluated based on the area under the curve (AUC). Genes with an AUC>0.7 were considered biomarkers demonstrating good diagnostic efficacy for IPF. The GSE24206 dataset was used as an external dataset to further clarify the expression of these hub genes in normal and IPF lung tissues.

#### 2.3.7 Single-cell data process and cluster

The raw single-cell RNA sequencing (scRNA-seq) data of the GSE271789 dataset were downloaded from the GEO database, containing of peripheral blood monocytes from 15 IPF patients and 15 healthy controls. The 10× scRNA-seq data were converted into Seurat objects using the ‘Seurat’ package of R software (version 5.0.0). The ‘FindVariableFeatures’ function was to filter the top 2000 hypervariable genes for quality control (QC). Principal component analysis (PCA) was performed on the top 2000 genes, and uniform manifold approximation and projection (UMAP) was used for dimensionality reduction and cluster identification. The ‘FindAllMarkers’ function was used to identify important marker genes. Comments based on cluster panglaodb (https://panglaodb.se/index.html) to download different cell types markers.

## 3 Results

### 3.1 Causal association of circulating immune cells with IPF

#### 3.1.1 Results of MR

**Figure 2:**
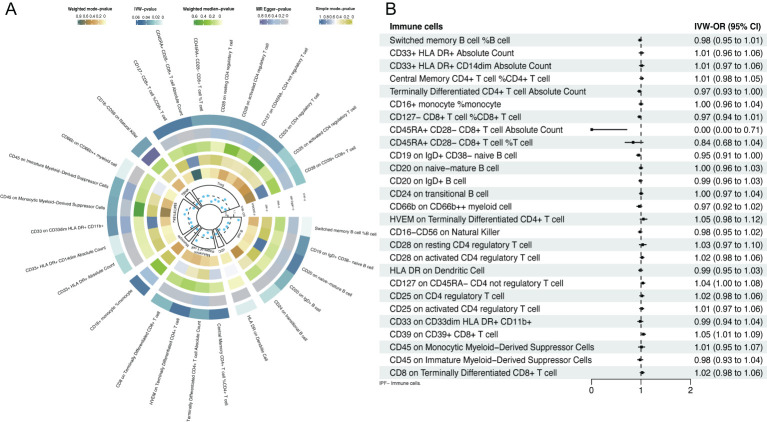
Forest plot of bidirectional MR; **(A)** causal effect of immune cells on IPF; **(B)** causal effect of IPF on immune cells.

IVs contained 1406 SNPs, all with an *F* value exceeding 10, indicating the absence of weak IVs (**Supplementary Table S1**). Twenty-seven immune cell phenotypes were identified through IVW, among which 20 were associated with a reduced risk of IPF and 7 were associated with an increased risk (**Figure 2A**). At the B cell level, switched memory B cells (*OR*=1.27, 95% *CI* [1.08-1.48], *P*=0.003) were associated with an increased risk of IPF. Conversely, CD19 on IgD^+^ CD38- naive B cells (*OR*=0.94, 95% *CI* [0.882-0.996], *P*=0.037), CD20 on naive mature B cells (*OR*=0.94, 95% *CI* [0.888-0.998], *P*=0.043), CD20 on IgD^+^ B cells (*OR*=0.93, 95% *CI* [0.86-0.99], *P*=0.028), and CD24 on transitional B cells (*OR*=0.81, 95% *CI* [0.69-0.96], *P*=0.013) were associated with a reduced risk of IPF development. At the DCs level, human leukocyte antigen (HLA) DR on DCs (*OR*=0.89, 95% *CI* [0.82-0.97], *P*=0.006) was associated with a reduced risk of IPF development. At the T cell level, CD8 on terminally differentiated CD8^+^ T cells (*OR*=1.18, 95% *CI* [1.02-1.37], *P*=0.027) was associated with an increased risk of IPF development. Conversely, central memory CD4^+^ T cells (*OR*=0.96, 95% *CI* [0.930-0.999], *P*=0.044), terminally differentiated CD4^+^ T cell absolute counts (*OR*=0.80, 95% *CI* [0.65-0.98], *P*=0.031), and HVEM on terminally differentiated CD4^+^ T cells (*OR*=0.92, 95% *CI* [0.852-0.998], *P=*0.046) were associated with a reduced risk of developing IPF. At the monocyte level, CD16^+^ monocytes (*OR*=0.86, 95% *CI* [0.738-0.996], *P*=0.045) were associated with a reduced risk of IPF development. At the myeloid level, CD45 on monocytic myeloid-derived suppressor cells (MDSCs) (*OR*=1.12, 95% *CI* [1.01-1.25], *P*=0.032) and CD45 on immature MDSCs (*OR*=1.08, 95% *CI* [1.01-1.17], *P*=0.030) were associated with an increased risk of IPF. Conversely, CD33^+^ HLA DR+ absolute counts (*OR*=0.94, 95% *CI* [0.88-0.99], *P*=0.016), CD33^+^ HLA DR^+^ CD14dim absolute counts (*OR*=0.92, 95% *CI* [0.87-0.98], *P*=0.006), CD33 on CD33dim HLA DR^+^ CD11b^+^ (*OR*=0.96, 95% *CI* [0.85-1.08], *P*=0.037), and CD66b on CD66b^++^ myeloid cells (*OR*=0.89, 95% *CI* [0.82-0.96], *P*=0.005) were associated with a reduced risk of IPF development. At the NK cells level, CD16^-^ CD56 on NK cells (*OR*=0.88, 95% *CI* [0.79-0.99], *P*=0.033) was associated with a reduced risk of IPF development. At the Tregs level, CD45RA^+^ CD28^-^ CD8^+^ T cell absolute counts (*OR*=1.001, 95% *CI* [1.001-1.002], *P*=0.044), CD45RA^+^ CD28^-^ CD8^+^ T cells (*OR*=1.001, 95% *CI* [ 1.00009-1.00219], *P*=0.033), and CD25 on CD4 Tregs (*OR*= 1.07, 95% *CI* [1.005-1.145], *P*=0.036) were associated with an increased risk of IPF development. Conversely, CD25 on activated CD4 Tregs (*OR*=0.80, 95% *CI* [0.67-0.97], *P*=0.020), CD28 on resting CD4 Tregs (*OR*=0.80, 95% *CI* [0.66-0.98], *P*=0.033), CD39 on CD39^+^ CD8^+^ T cells (*OR*=0.87, 95% *CI* [0.77-0.98], *P*=0.024), CD127 on CD45RA^-^ CD4 not Tregs (*OR*=0.89, 95% *CI* [0.79-0.99], *P*=0.036), CD127^-^ CD8^+^ T cell %CD8^+^ T cell (*OR*= 0.888, 95% *CI* [0.791-0.997], *P*=0.044), and CD28 on activated CD4 Tregs (*OR*=0.89, 95% *CI* [0.81-0.99], *P*=0.035) were associated with a reduced risk of IPF development. Additionally, the MR-Egger, weighted median, simple mode, and weighted mode results were generally consistent with the IVW results, indicating that the results were reliably robust.

Inverse MR analysis indicated that IPF was only causally associated with certain immune cell phenotypes, including CD45RA*^+^* CD28*^-^* CD8*^+^* T cell absolute counts (*OR*=0.001, 95% *CI* [0.00-0.71], *P*=0.037), CD19 on IgD*^+^* CD38*^-^* naive B cells (*OR*=0.95, 95% *CI* [ 0.909-0.998], *P*=0.042), and CD39 on CD39*^+^* CD8*^+^* T cells (*OR*=1.05, 95% *CI* [1.01-1.09], *P*=0.012; **Figure 2B**, **Supplementary Table S2**).

#### 3.1.2 Sensitivity analysis

Cochrane's *Q* test revealed heterogeneity among the 27 SNPs for CD16^-^CD56 on NK cells (*P*=0.020), whereas no heterogeneity was observed among the SNPs for the remaining 26 immune cell phenotypes. The MR-Egger intercept indicated no horizontal pleiotropy between the SNPs of the 27 immune cell phenotypes (*P*>0.050). MR-PRESSO results showed outliers in CD16^-^ CD56 on NK cells, and even after correcting for these outliers, heterogeneity persisted in CD16^-^ CD56 on NK cells (*P*=0.018; **Table 1**). The results of the IVW remained largely unchanged after removing individual SNPs in LOO analysis, suggesting robust sensitivity (**Supplementary Figure 1**).

**Table 1:** Sensitivity analysis of MR results.

GWAS ID	Exposure	Cochrane's Q test pval	MR-Egger intercept pval	MR-PRESSO global pval (Outlier corrected pval)
ebi-a-gcst90001884	CD16^-^CD56 on NK	0.020	0.230	0.034 (0.018)
ebi-a-gcst90001762	CD20 on IgD^+^ B cell	0.270	0.960	0.176
ebi-a-gcst90002106	HLA DR on DCs	0.980	0.550	0.302
ebi-a-gcst90001545	Terminally Differentiated CD4^+^ T cell absolute count	0.130	0.910	0.369
ebi-a-gcst90001936	CD25 on CD4 regulatory T cell	0.370	0.340	0.379
ebi-a-gcst90001902	CD28 on activated CD4 regulatory T cell	0.710	0.720	0.389
ebi-a-gcst90001836	CD66b on CD66b^++^ myeloid cell	0.840	0.090	0.421
ebi-a-gcst90002052	CD45 on Immature MDSCs	0.540	0.420	0.422
ebi-a-gcst90002029	CD39 on CD39^+^ CD8^+^ T cell	0.510	0.960	0.432
ebi-a-gcst90001923	CD127 on CD45RA^-^ CD4 not regulatory T cell	0.190	0.200	0.438
ebi-a-gcst90001727	CD19 on IgD^+^ CD38^-^ naive B cell	0.260	0.550	0.444
ebi-a-gcst90001939	CD25 on activated CD4 regulatory T cell	0.620	0.760	0.467
ebi-a-gcst90001759	CD20 on naive^-^ mature B cells	0.370	0.490	0.545
ebi-a-gcst90001698	CD45RA^+^ CD28^-^ CD8+ T cell absolute count	0.740	0.100	0.551
ebi-a-gcst90001538	Central Memory CD4+ T cell %CD4^+^ T cell	0.500	0.280	0.585
ebi-a-gcst90001517	CD33^+^ HLA DR^+^ Absolute Count	0.870	0.660	0.651
ebi-a-gcst90001683	CD127^-^ CD8^+^ T cell %CD8^+^ T cell	0.710	0.870	0.653
ebi-a-gcst90001948	CD33 on CD33^dim^ HLA DR^+^ CD11^b+^	0.240	0.490	0.694
ebi-a-gcst90001879	HVEM on terminally differentiated CD4^+^ T cell	0.790	0.420	0.713
ebi-a-gcst90001900	CD28 on resting CD4 regulatory T cell	0.680	0.960	0.736
ebi-a-gcst90002049	CD45 on monocytic MDSCs	0.790	0.760	0.737
ebi-a-gcst90001774	CD24 on transitional B cell	0.360	0.390	0.750
ebi-a-gcst90002057	CD8 on terminally differentiated CD8^+^ T cell	0.830	0.540	0.813
ebi-a-gcst90001402	Switched memory B cell %B cell	0.810	0.380	0.826
ebi-a-gcst90001700	CD45RA^+^ CD28^-^ CD8^+^ T cell %T cell	0.400	0.990	0.858
ebi-a-gcst90001587	CD16^+^ monocyte %monocyte	0.410	0.780	0.890
ebi-a-gcst90001520	CD33^+^ HLA DR^+^ CD14^dim^ absolute count	0.530	0.850	0.979

Moreover, no heterogeneity, outliers, or horizontal pleiotropy was observed among the SNPs included in the reverse MR analysis (*P*>0.050; **Supplementary Table S3**). LOO analysis also demonstrated good sensitivity (**Supplementary Figure 2**).

### 3.2 Analysis of transcriptomics data

#### 3.2.1 DEGs screening and immune cell infiltration

**Figure 3:**
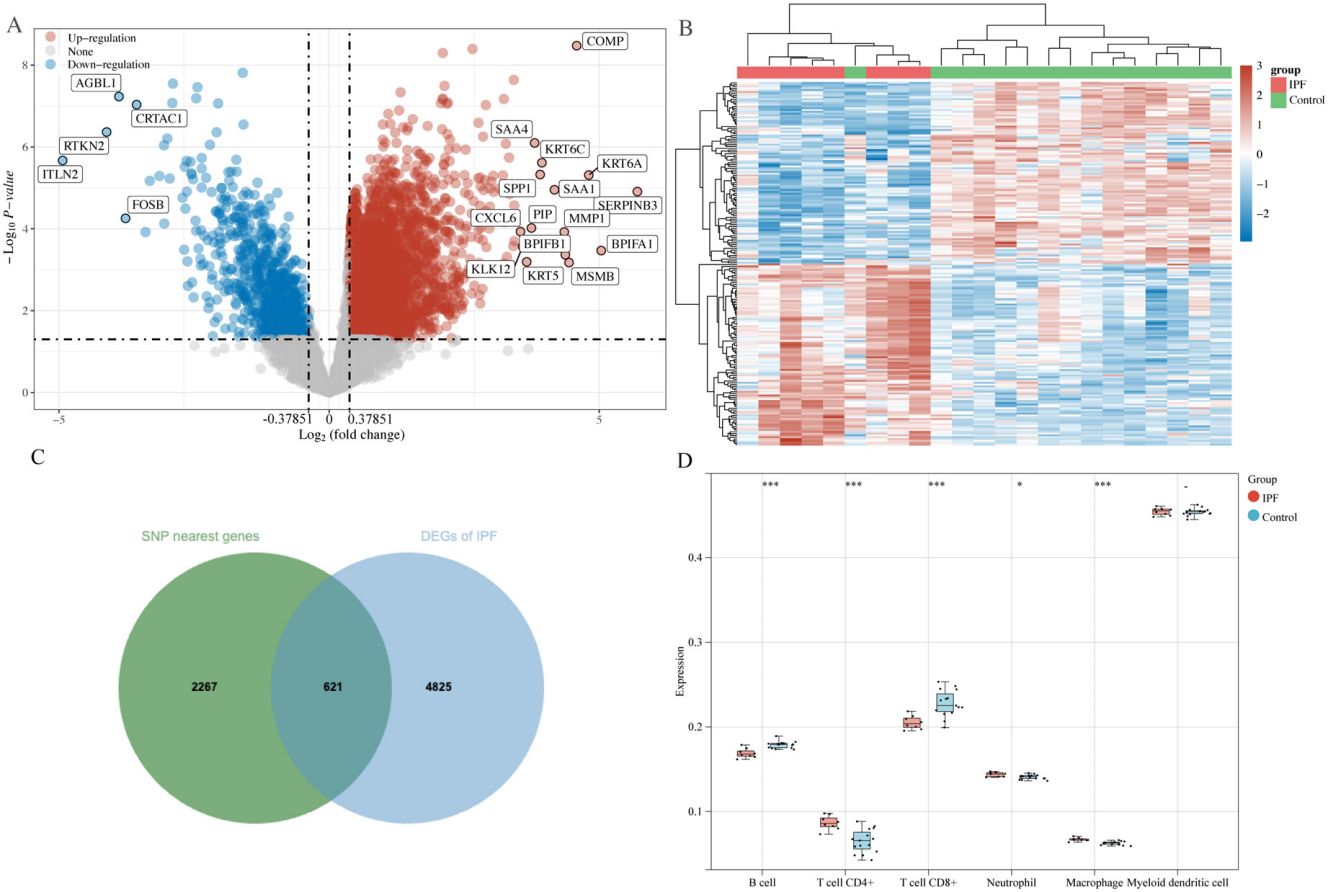
DEGs screening and immune cell infiltration analysis. **(A)** Volcano plot: screening of DEGs in IPF; **(B)** heatmap: expression of DEGs in control and IPF groups; **(C)** Venn diagram: screening of SNP nearest genes and shared genes of DEGs; **(D)** Timer algorithm reveals the infiltration of five types of immune cells by immune infiltration analysis. *P<0.05, ***P<0.001.

In total, 2888 SNP-nearest genes were obtained through the GWAS catalog database. From the GSE10667 dataset, 5446 DEGs of IPF were identified, with 4594 upregulated and 852 downregulated DEGs in IPF (**Figures 3A, B**). Finally, 621 DEGs overlapped with SNP-nearest genes (**Figure 3C**). The results of immune cell infiltration analysis using the TIMER algorithm revealed significant correlations between the expression of these 621 genes and the degree of B cell, CD4^+^ T cell, CD8^+^ T cell, neutrophil, and macrophage infiltration (**Figure 3D**).

#### 3.2.2 WGCNA, enrichment and PPI analysis

**Figure 4:**
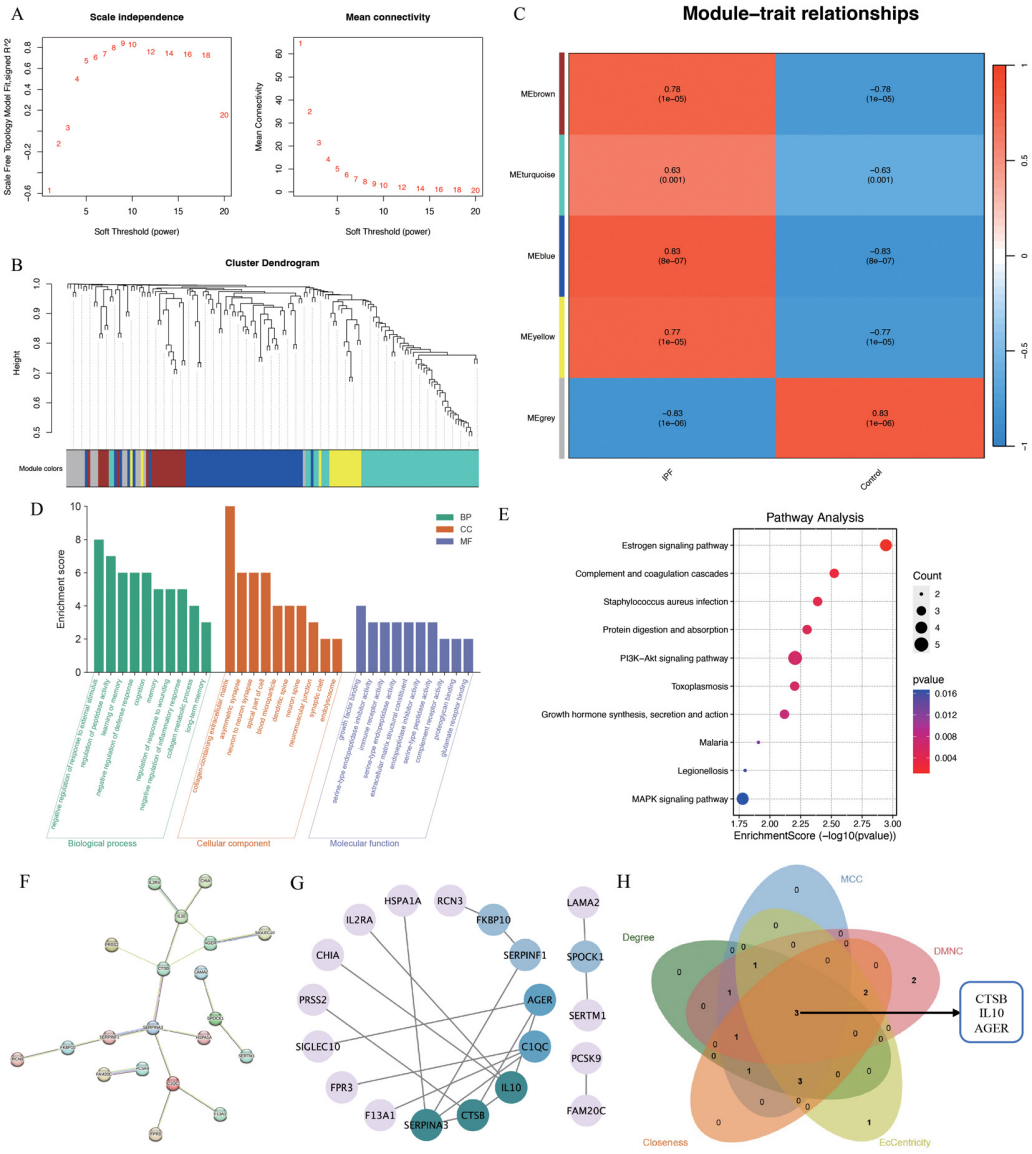
WGCNA analysis of IPF-related genes. **(A)** Scale independence; **(B)** independent concatenation analysis identifying the soft threshold β as 10; **(C)** module-trait correlation; **(D)** Go enrichment analysis; **(E)** KEGG pathway analysis; **(F, G)** PPI analysis; **(H)** venn plot of the five core algorithms to obtain hub genes.

To further elucidate IPF core genes, WGCNA was applied to the 621 shared genes to identify gene modules with the highest correlation with IPF. A soft threshold of 10 was set (**Figure 4A**). In total, five gene modules were identified (**Figure 4B**), with the blue module showing the highest correlation with IPF (r=0.83, *P*=8e-07; **Figure 4C**). The blue module genes were primarily enriched in the negative regulation of response to external stimulus, regulation of peptidase activity, learning or memory, and negative regulation of defense response in biological processes according to GO enrichment. In terms of cellular components, these genes were predominantly enriched in the collagen-containing extracellular matrix (ECM), asymmetric synapse, neuron-to-neuron synapse, and apical part of the cell. In terms of molecular functions, the genes were mainly enriched in growth factor binding, serine-type endopeptidase inhibitor activity, immune receptor activity, and serine-type endopeptidase activity (**Figure 4D**). KEGG pathway enrichment indicated that the blue module genes were enriched in signaling pathways such as the estrogen, PI3K-Akt, and MAPK signaling pathways (**Figure 4E**). The PPI network identified 20 reciprocal genes (**Figures 4F, G**). Using the Cytohubba plug-in, cathepsin B (*CTSB*), interleukin 10 (*IL10*), and advanced glycosylation end-product specific receptor (*AGER*) were identified as core genes (**Figure 4H**).

#### 3.2.3 Diagnostic marker identification and external dataset validation

**Figure 5:**
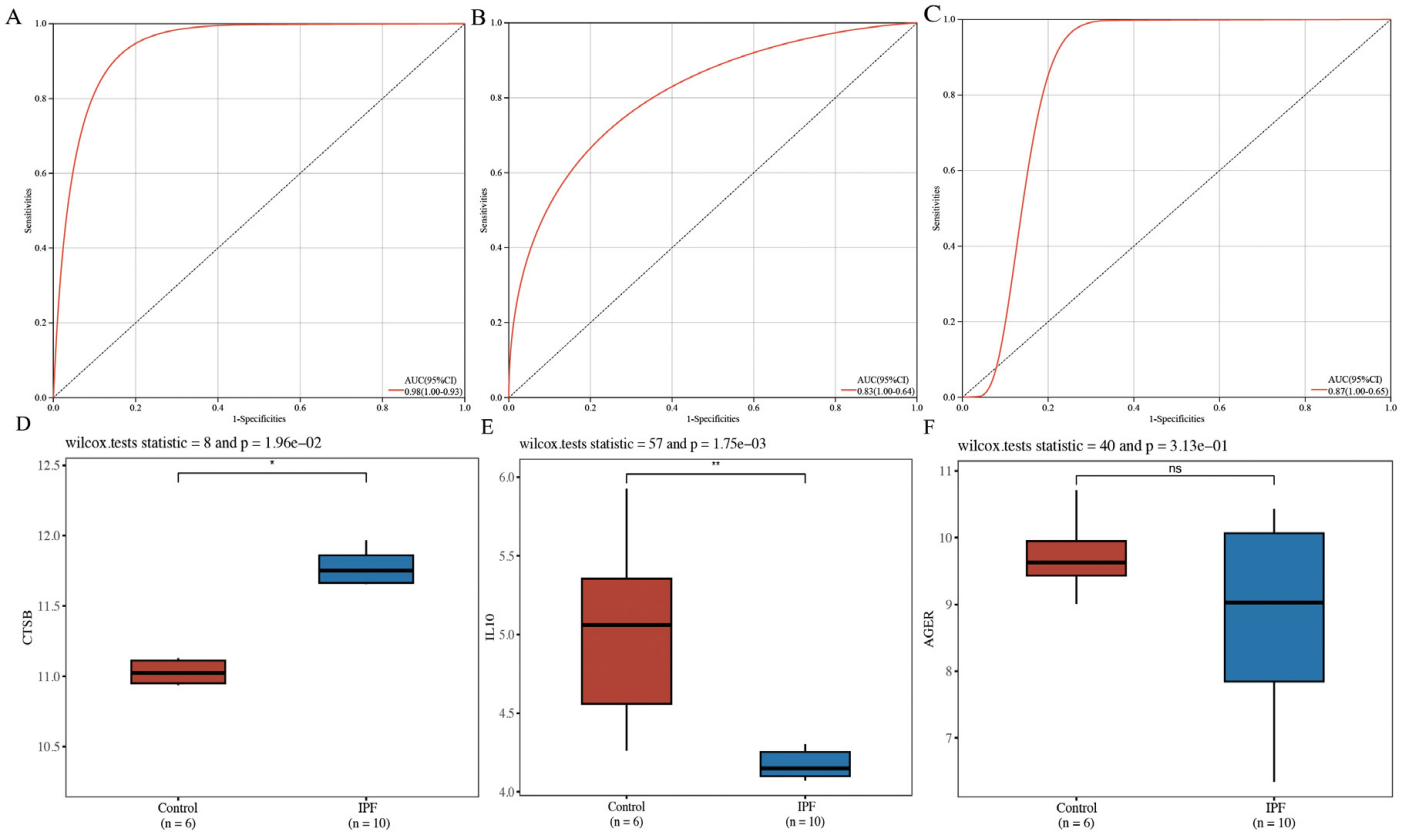
Diagnostic efficacy determination and validation of hub genes. **(A)** ROC curve for CTSB; **(B)** ROC curve for IL10; **(C)** ROC curve for AGER; **(D)** validation of external dataset for CTSB (GSE24206); **(E)** validation of external dataset for IL10 (GSE24206); **(F)** validation of external dataset for AGER (GSE24206). *P<0.05, **P<0.01, ns P>0.05.

ROC curves were plotted for *CTSB*, *IL10*, and *AGER*, demonstrating that *CTSB* (AUC=0.98), *IL10* (AUC=0.83), and *AGER* (AUC=0.87) effectively distinguished IPF from controls, indicating their potential diagnostic markers for IPF (**Figures 5A–C**). Validation of the expression of these hub genes in IPF was conducted using GSE24206 as an external dataset. Results indicated a significant elevation in the expression of *CTSB* in IPF lung tissues (*P*=0.020), whereas the expression of *IL10* was significantly decreased (*P*=0.002) (**Figures 5D, E**). There was no significant difference in AGER expression (*P*=0.313) (**Figure 5F**).

### 3.3 Identification of hub genes in scRNA-seq

**Figure 6:**
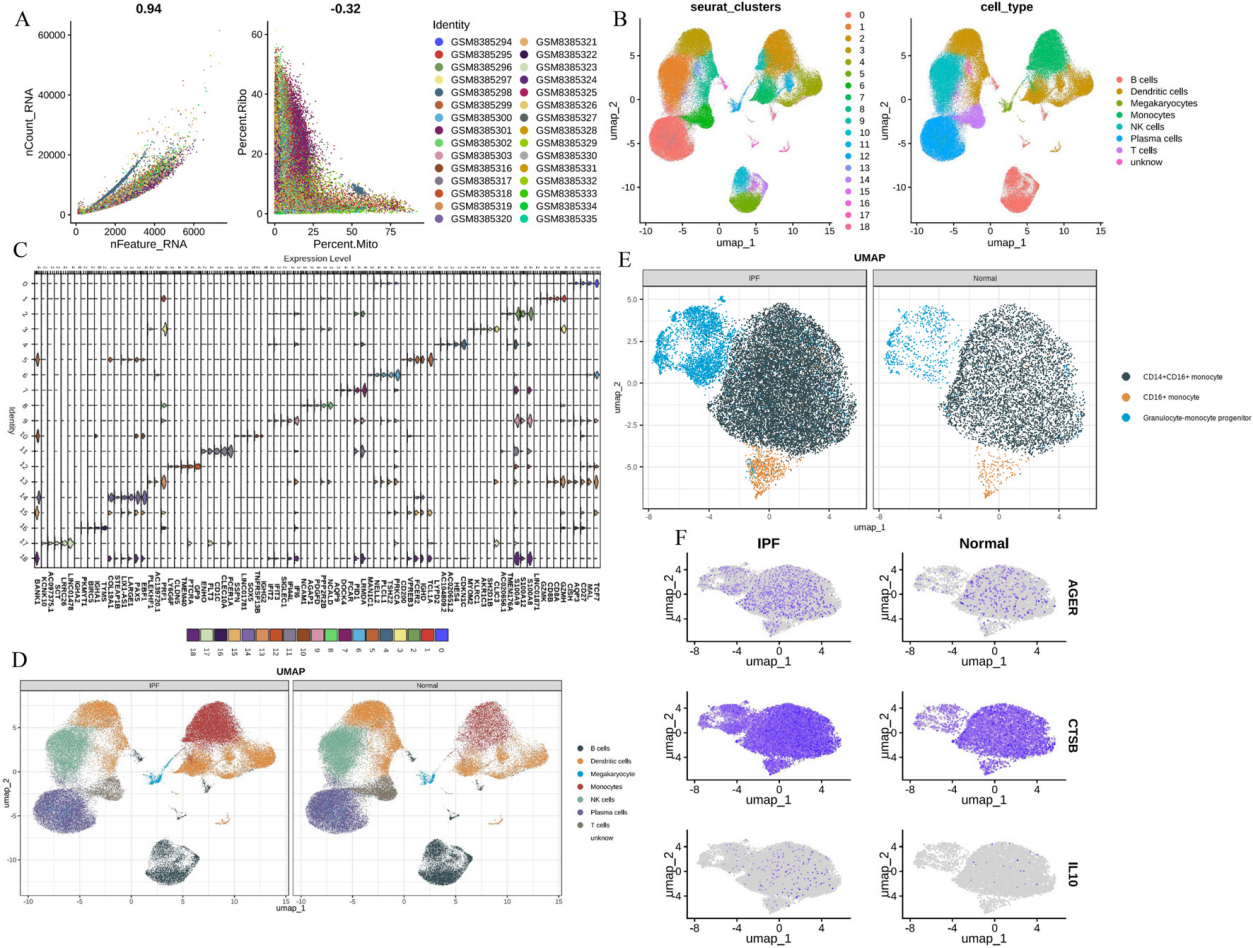
Single-cell analysis **(A)** The QC of total scRNA - seq data; **(B)** UMAP revealed 19 clusters under a resolution of 0.4; **(C)** marker genes of 19 clusters; **(D)** eight cell types were generated and colored; **(E)** identification of three monocytes phenotypes; **(F)** CTSB, IL10, AGER expression levels in monocytes.

To further validate the MR Results and the expression of hub genes, single-cell analysis was performed. First, we performed QC (**Figure 6A**). Post-QC cells were dimension-reduced using UMAP and divided into 19 clusters (**Figure 6B**), annotating as 8 immune cell types, including ‘B cells’, ‘DCs’, ‘Megakaryocytes’, ‘Monocytes’, ‘NK cells’, ‘Plasma cells’, ‘T cells’ and ‘unknown’. Marker genes were identified for each cell type (**Figure 6C**). The immune cells in peripheral blood monocytes of patients with IPF are different from those of healthy controls, especially monocytes (**Figure 6D**), including alveolar macrophages and other immune cells. We further characterized monocytes into three subsets, namely CD14^+^ CD16^+^ monocytes, CD16^+^ monocytes and Granulocyte-monocyte progenitor. The distribution of these three monocyte subsets was significantly higher in patients with IPF than in healthy controls (**Figure 6E**). Notably, CTSB, IL10, and AGER were all expressed higher in monocytes from IPF patients than in healthy controls, highlighting the potential feasibility of these three hub genes as biomarkers (**Figure 6F**).

## 4 Discussion

In this study, we performed a two-sample, bidirectional MR study to assess the causal association between 731 circulating immune cell phenotypes and IPF. Genetic variants were used as probes in the forward MR analysis, revealing 27 immune cell phenotypes causally associated with IPF. Among these, 20 were associated with a reduced risk of developing IPF, whereas 7 were associated with an increased risk of developing IPF. In the reverse MR analysis, IPF was found to be causally associated with three immune cell phenotypes. Quality control assessment demonstrated the stability of the MR findings. By combining MR with transcriptomics data to explore genetic causality, we performed pathway enrichment analyses and identified three markers: *CTSB*, *IL10*, and *AGER*. These markers can serve as causal diagnostic indicators of immune cell-IPF interactions, offering valuable insights into the validation of a cohort of IPF patients.

Immune cells are involved in nearly all wound healing processes and immune-inflammatory responses. In IPF, a disease of localized wound healing and fibrosis in the lungs, dysregulated immune responses play a crucial role in its pathogenesis and progression (18). In the present study, we focused on circulating immune cells, identifying seven immune cell populations (Tregs, myeloid cells, B cells, maturation stages of T cells, monocytes, NK cells, and cDC) causally involved in IPF. Immune infiltration analysis of SNP-nearest genes jointly with transcriptomics data demonstrated that genes could influence IPF by modulating immune cells, thus validating the MR results. Among these, Tregs, a key subset of helper T cells, exhibited the most pronounced correlation with IPF in our study. Prior research has shown that Tregs are involved in various respiratory diseases and play a bidirectional regulatory role in the development of IPF. They can mitigate fibrosis by blocking the upstream inflammatory stress process, thus inducing immunosuppression. Conversely, they secrete TGF-β and platelet-derived growth factor-B, thereby triggering the fibrotic process (19). This may be attributed to the functional heterogeneity of the Tregs subpopulations, characterized by differential expression of surface antigens (18). A cohort study (20) has demonstrated a significant characteristic disorder of Tregs subpopulations in the peripheral blood of IPF patients, with the proportion of activated Tregs subpopulations inversely correlated with disease severity and progression. Our results revealed an overall negative regulatory effect of Tregs subpopulations on IPF. These encompassed three Tregs subpopulations and their specific surface antigens (CD45RA^+^ CD28^-^ CD8^+^ T cell absolute counts, CD45RA^+^ CD28^-^ CD8^+^ T cell %T cell, and CD25 on CD4 Tregs) that positively regulate IPF, corroborating findings from previous studies. Reverse MR analysis of this manuscript revealed a regulatory effect of IPF on three subpopulations of Tregs (CD39 on CD39^+^ CD8^+^ T cells, CD45RA^+^ CD28^-^ CD8^+^ T cell absolute counts, and CD127 on CD45RA^-^ CD4 not Tregs). This finding suggests that as the disease progresses with IPF, it may induce regulation of the proportion of certain Tregs subpopulations.

Myeloid cells, including macrophages, monocytes, DCs, and granulocytes, are commonly used to measure tissue infections and injuries. They are ubiquitous throughout the body and play a crucial role in the formation and development of lung tissue structure, ECM, and blood vessels as the first responders of immune response (21, 22). Single-cell sequencing studies of IPF lung tissues have revealed that the highly expressed MerTK receptor on the surface of lung macrophages mediates efferocytosis and drives the development of IPF (23). Infiltrating monocytes inhibit lung fibroblast activation, viability, and migration by regulating IL10 (24). DCs serve as a bridge between innate and adaptive immunity. DCs-rich lymphoid aggregates can be found in lung tissues of IPF patients, and DCs may participate in the degradation of collagen and related proteins in fibrosis by upregulating matrix metalloproteinases (MMPs) in IPF tissues (25). Furthermore, the flow cytometry in blood samples of IPF patients has revealed a significant reduction in all blood subtypes of DCs, and their role in IPF needs further elucidation (26). In our study findings, all positive myeloid cells exhibited the distinct cellular markers of ‘CD' to recognize antigen. MDSCs and monocytes with CD45^+^ markers were positively causally associated with IPF, whereas HLA DR^+^ cells with CD33 and CD66^+^ positive MDSCs were negatively causally associated with IPF. MDSCs, a heterogeneous population of immature myeloid cells (including myeloid progenitor cells, immature monocytes or DCs, or immature granulocytes) with potent suppressive capacity, are associated with IPF (27). Their recruitment markedly increases in the circulating and lung tissues and contributes to the disease through their paracrine roles in promoting myofibroblast differentiation, immunosuppression, and other processes (27, 28). Our results demonstrate that the CD45/CD66 signature of MDSCs may serve as a cellular marker for the bidirectional regulation of IPF and affirm the possible role of CD45^+^ monocytes and CD66 differentiation-associated neutrophils in IPF. Genetic variants in the HLA region, also known as the major histocompatibility complex region, have been associated with inflammation and respiratory diseases and play a similarly important role in IPF (29). The role of CD33 as an important cellular marker in IPF for MDSCs and mature myeloid cells has been emphasized several times in both previous studies and the current one. However, while previous studies demonstrate a positive promotional effect of CD33 for IPF (30), our results indicated that cellular expression of CD33 may negatively regulate IPF. Thus, further investigation is warranted on the role of CD33 in the regulation of IPF. Similarly, we found an inverse association of CD16+ monocytes with IPF disease progression on MR analysis, whereas previous studies (31) and our single-cell analysis have highlighted their disease-promoting role, with CD16+ monocyte subsets preferentially interacting with endothelial cells. It may promote the healing of early inflammation and tissue repair through myofibroblast accumulation, angiogenesis, and collagen deposition (28). However, its role in IPF is still unclear, and it may be a specific precursor of monocyte-macrophages in the lung interstitium, which deserves further discussion (32).

B cells, integral players in the adaptive immune response, differentiate into plasma cells that produce antibodies in response to antigenic stimulation through the germinal center response (33). B cells are enriched in IPF lung tissues (13), and circulating B cell-activating factors and B cell chemotactic mediator CXCL13 are highly expressed in IPF patients, suggesting a pivotal role for B cells in IPF progression. We identified five B cell phenotypes (CD24 on transitional B cells, CD19 on lgD^+^ B cells, CD20 on lgD^+^ B cells, CD20 on naive mature B cells, and switched memory B cells) causally associated with IPF. Among them, switched memory B cells were associated with an increased risk of IPF. Transitional and naive mature B cells, representing relatively early stages of B cell differentiation, expressed IgD molecules on their surfaces, with IgD expression peaking at the naive mature B cell stage. In contrast, switched memory B cells, representing a late stage of B cell differentiation, did not express IgD molecules on their surface. Therefore, we hypothesized that IgD^+^ B cells may be a protective factor against IPF. Studies have shown that IgD may be associated with B cell development and mucosal immunity (34). IgD exists in transmembrane and secreted forms. The transmembrane form is highly expressed on the surface of human mature B cells, and the secreted form is released into sites such as blood, respiratory tract, and saliva (35). Increased levels of IgD have been observed in the serum of patients with asthma and COPD, suggesting a potential association of IgD with chronic inflammation of the lower respiratory tract (36). However, the role of IgD in IPF remains poorly understood. IgD^-^ B cells are expressed in the blood of patients with COVID-19 and correlate with tissue inflammation and fibrosis development in autoimmune fibrotic diseases, while IgD^+^ B cells are highly expressed in patients with hereditary pulmonary fibrosis (37, 38).

Enrichment analysis showed that immune cells may play an important role in the regulation of peptidase activity through negative regulation of response to external stimulus, regulation of peptidase activity, learning or memory and negative regulation of defense response are involved in the pathogenesis of IPF. Immune cell disorder is an important part of the process of IPF, which is regulated by inflammatory crosstalk after epithelial cells and endothelial cells are damaged. Immunometabolic reprogramming caused by immune cells further interferes with the metabolic process of the body and participates in IPF. For example, T cells may regulate the biosynthesis of collagen by participating in amino acid metabolism and other processes to promote the formation of IPF (39). Immune cells exert mechanical stress and force on the surrounding environment, participate in the shaping of microenvironment structure and long-term material exchange, activate gene expression and exert regulation. Although little is known, it is clear that immune synapses participate in this physicochemical process and play an important role (40). Immune cells drive growth factor binding to participate in angiogenesis, wound healing, cell migration and EMT, and then participate in the process of IPF (41). IPF has obvious gender differences, and estrogen signaling pathway has been reported to play an important role in a variety of PF models (42). Alveolar macrophages have estrogen receptor specificity. PI3K-Akt and MAPK signaling pathway are classical pathways, which interfere with IPF mainly by participating in EMT (43, 44), autophagy (45, 46) and other pathways.

CTSB is one of the 11 cysteine histone (histones B, H, L, S, C, K, O, F, V, X, and W) members of the human genome, primarily involved in the lysosomal terminal degradation of endocytosed proteins (47). Additionally, it is a potent ECM-degrading enzyme involved in lung homeostasis. Monocyte-derived macrophages synthesize CTSB in response to chronic inflammation and process it into a mature enzyme. CTSB is involved in the pathophysiological mechanisms of ECM remodeling (48). It shows a correlation between the expression of TGF-β1 and α-SMA, which can be involved in TGF-β1-driven fibroblastic cellular differentiation in IPF (49). Its expression level in serum is closely correlated with the progression of IPF (50).

IL10 is a potent immune cell suppressor involved in innate and adaptive immunity and associated with various immune cells, including Tregs, CD8^+^ T cells, DCs, macrophages, mast cells, NK cells, eosinophils, and neutrophils (51). The specific loss of IL10 in B cells can lead to increased expression of proinflammatory cytokines, persistent leukocyte infiltration and prolonged alveolar barrier damage in a mouse model of acute lung injury (52). At the same time, it can also be found that the expression of CCL-20 in alveolar epithelium and the recruitment of DCs are reduced in a mouse model of asthma, thereby inhibiting the allergic state (53). It suggests a close relationship between B cells and IL10 in lung disease progression. A study found reduced IL10 expression in IPF (54), which is consistent with our externally validated results. IL10 can attenuate fibrosis in the early stages of inflammation by affecting fibroblast activation, viability, and migration while exhibiting anti-PF effects by inhibiting TGF-β1 expression (24, 55).

AGER, also known as RAGE, is a transmembrane protein receptor belonging to the immunoglobulin superfamily involved in innate and adaptive immune processes. Exposure of AGER to the surface of macrophages or other immune cells can trigger iron death, leading to the release of proinflammatory cytokines (56). The soluble receptor for advanced glycosylation end products (sRAGE) is thought to act as a decoy to capture AGER. AGER targeted inhibition can improve a variety of lung diseases (57–59). Significantly elevated genetic polymorphisms of AGER and elevated sRAGE can be found in patients with IPF. These factors have been associated with acute exacerbation of IPF (60–62) and are considered primary influences on the five-year survival of IPF patients. AGEs are the primary AGER conjugates and serve as biomarkers of the aging process *in vivo*. They are involved in several processes such as collagen deposition, TGF-β1 synthesis, EMT, cytotoxicity, and death. AGEs are predominantly present in type I alveolar epithelial cells and associated with type II alveolar epithelial differentiation into type I alveolar epithelial cells, lung development, reepithelialization, and maintenance of epithelial adherence to basement membranes (63–65). AGER, as a systemic inflammatory marker, exhibits abnormal expression in IPF and is often accompanied by elevated expression of MMP1 and MMP7, a phenomenon that may be associated with type I alveolar epithelial cells (66, 67). Although no significant differential expression of AGER was found in the validation of an external cohort dataset, its involvement in the immune-related processes of IPF warrants further investigation.

Previous studies have suggested that these three genes may be important in the process of IPF, our study used MR combined with more bioinformatics methods to demonstrate that these three genes are involved in the process of IPF treated by immune cells and are good biomarker candidates.

Although this study was based on the latest and largest GWAS and used rigorous statistical methods, it still has some limitations: (1) As most publicly available GWAS data are based on the European population, the bias of the study results cannot be completely avoided considering population stratification factors and may limit the generalizability of the results. In the future, data from a larger sample size with more populations should be considered for validation of the results. (2) The GWAS statistics used in the study are general and do not include the personal data of the participants, such as underlying diseases, living habits, and dietary preferences. These factors may give rise to potential non-linear relationships that are beyond the control of the study and cannot be clarified through subgroup analysis. (3) The severity of the disease was not taken into account, and different stages and degrees of disease development may have an impact on the results, affecting their reliability. (4) Although we have elucidated the causal relationship between immune cells and IPF based on the current research technology, we cannot deny that the interaction among genetics, disease, and environment is extremely complex. There may be deeper influences and interactions that interfere with the reliability of the results. Therefore, further research is still needed to confirm the causal relationship between immune cells and IPF.

## 5 Conclusion

Our findings highlight bidirectional causality, potential shared pathways, and genetic interactions between circulating immune cells and IPF. These new genetic insights may be crucial for understanding the pathogenesis of IPF and could offer novel perspectives for developing IPF-targeted therapies.

## Data Availability

The original contributions presented in the study are included in the article/**Supplementary Material**. Further inquiries can be directed to the corresponding author.
