# Supplementary figures and images for: Elucidating the causal associations and mechanisms between circulating immune cells and idiopathic pulmonary fibrosis: new insights from Mendelian randomization and transcriptomics
Source: Front Immunol. 2025 Jan 17;15:1437984. doi: 10.3389/fimmu.2024.1437984 (PMC11782250; doi:10.3389/fimmu.2024.1437984)

**Supplementary figures**

**
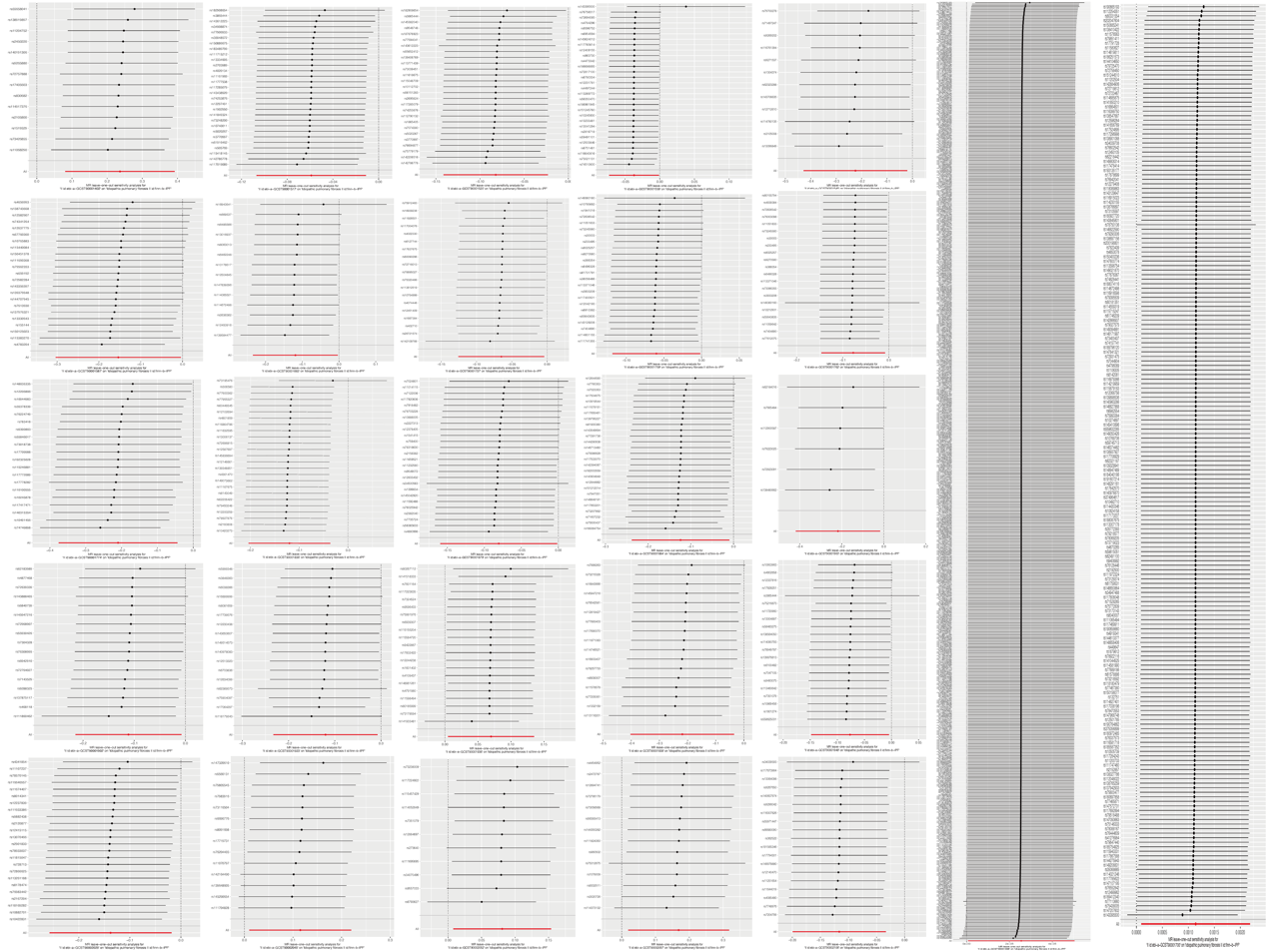
**

**Figure 1** LOO of immune cells to IPF

**
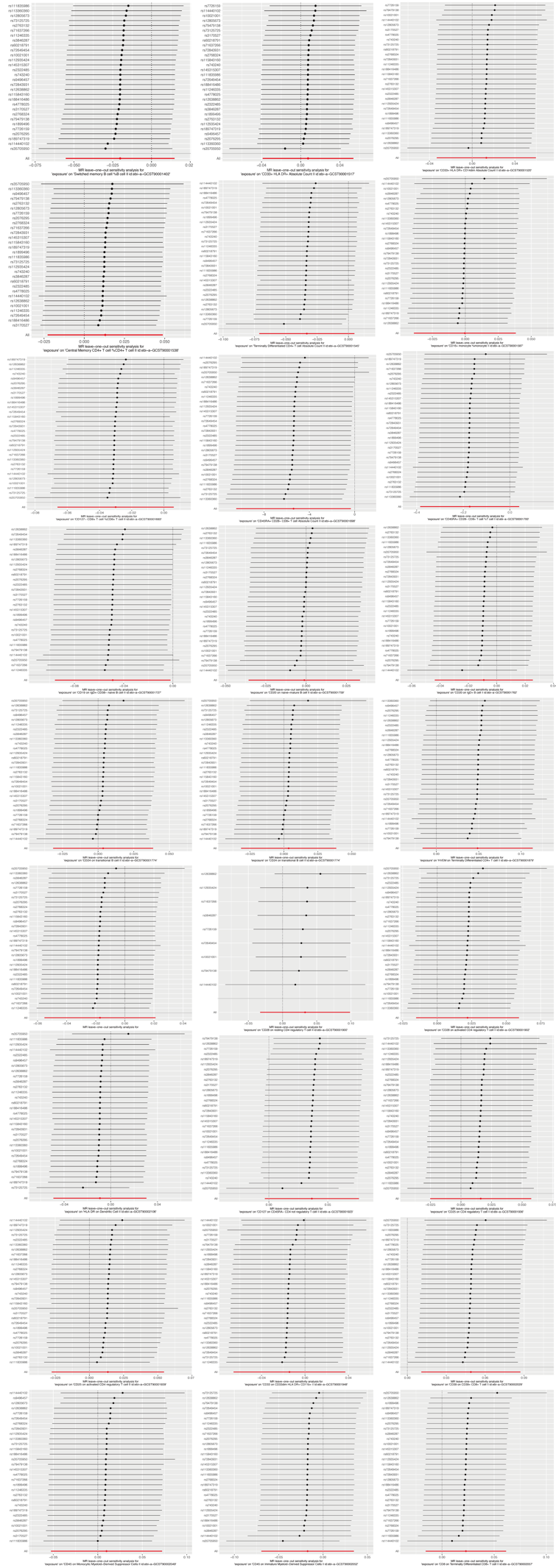
**

**Figure 2** LOO of IPF to immune cells

Supplement: Supplementary file 1 [file DataSheet1.docx]
